# Sexual versus Asexual Reproduction: Distinct Outcomes in Relative Abundance of Parthenogenetic Mealybugs following Recent Colonization

**DOI:** 10.1371/journal.pone.0156587

**Published:** 2016-06-20

**Authors:** Jun Tabata, Ryoko T. Ichiki, Hirotaka Tanaka, Daisuke Kageyama

**Affiliations:** 1 National Institute for Agro-Environmental Sciences, 3-1-3 Kannondai, Tsukuba, Ibaraki 305–8604, Japan; 2 Japan International Research Center for Agricultural Sciences, 1–1 Ohwashi, Tsukuba, Ibaraki 305–8686, Japan; 3 Tottori Prefectural Museum, 2–124 Higashi-machi, Tottori, Tottori 680–0011, Japan; 4 National Institute of Agrobiological Sciences, 1–2 Ohwashi, Tsukuba, Ibaraki 305–8634, Japan; University of Poitiers, FRANCE

## Abstract

Asexual reproduction, including parthenogenesis in which embryos develop within a female without fertilization, is assumed to confer advantages over sexual reproduction, which includes a “cost of males.” Sexual reproduction largely predominates in animals, however, indicating that this cost is outweighed by the genetic and/or ecological benefits of sexuality, including the acquisition of advantageous mutations occurring in different individuals and the elimination of deleterious mutations. But the evolution of sexual reproduction remains unclear, because we have limited examples that demonstrate the relative success of sexual lineages in the face of competition from asexual lineages in the same environment. Here we investigated a sympatric occurrence of sexual and asexual reproduction in the pineapple mealybug, *Dysmicoccus brevipes*. This pest invaded southwestern Japan, including Okinawa and Ishigaki Islands, in the 1930s in association with imported pineapple plants. Our recent censuses demonstrated that on Okinawa sexually reproducing individuals can coexist with and even dominate asexual individuals in the presence of habitat and resource competition, which is considered to be severe for this nearly immobile insect. Molecular phylogeny based on partial DNA sequences in the mitochondrial and nuclear genomes, as well as the endosymbiotic bacterial genome, revealed that the asexual lineage diverged from a common sexual ancestor in the relatively recent past. In contrast, only the asexual lineage exhibiting obligate apomictic thelytoky was discovered on Ishigaki. Co-existence of the two lineages cannot be explained by the results of laboratory experiments, which showed that the intrinsic rate of increase in the sexual lineage was not obviously superior to that of the asexual lineage. Differences in biotic and/or abiotic selective forces operating on the two islands might be the cause of this discrepancy. This biological system offers a unique opportunity to assess the relative success of sexual versus asexual lineages with an unusual morphology and life cycle.

## Introduction

Asexual reproduction, in which offspring arise from a single female organism, occurs in a variety of eukaryotes including plants, fungi, and animals. It is assumed to confer some advantages over a sexual reproduction, in which individuals of two genders, females and males, must be involved but only females can give birth to new individuals [1–10]. Despite the assumption that there is a “cost of males” in sexual reproduction, this system largely predominates in animals, indicating that this cost is outweighed by the benefits of sexual reproduction [1–3,7–9]. Although a variety of theories have been proposed to explain the genetic and ecological fitness associated with sexual reproduction, the evolution of sexuality and reproductive systems remains as one of the major unresolved puzzles in biology [4]. In particular, there are few examples that demonstrate the success and persistence of sexual lineages in the face of competition from asexual lineages in natural environments [11,12].

**Fig 1:**
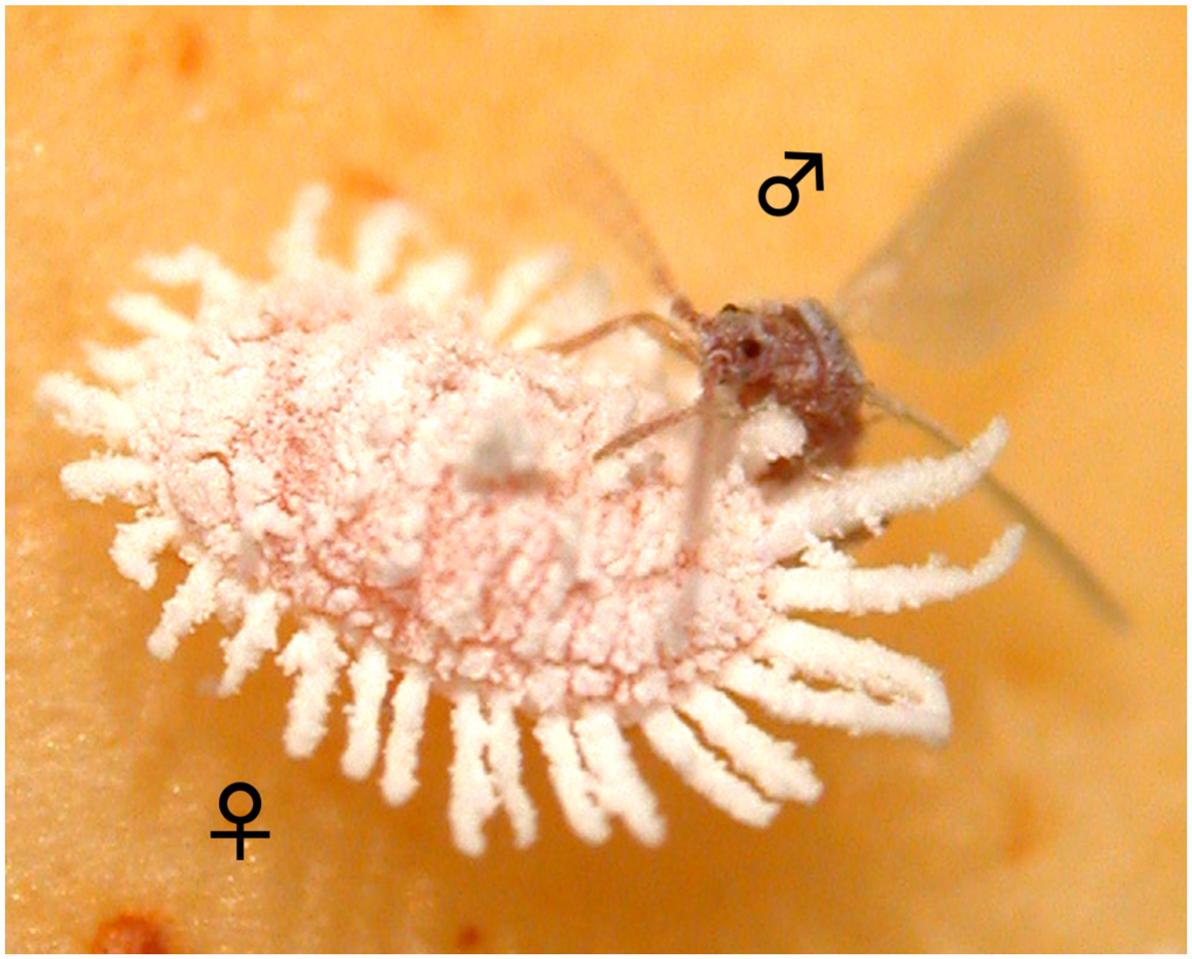
Copulation of the pineapple mealybug, *Dysmicoccus brevipes*. Adult males and females exhibit completely different appearances and biology.

Scale insects (Insecta: Hemiptera: Coccoidea), which include mealybugs (Pseudococcidae), are interesting for studies of the evolution and ecology of sexuality and asexuality, because this taxon exhibits various systems that control sex determination, sexual development, sex ratio, and mode of reproduction [13,14]. Such an extraordinary diversity of genetic systems is considered to be, at least partly, associated with their unusual morphology (Fig 1) and life cycle [13,15]. Scale insects are plant sap feeders closely related to aphids and whiteflies and are characterized by their unusual shapes. Adult females show development with retention of juvenile physical characters (neoteny) and are relatively immobile, lacking wings and often even legs. They produce body-covering secretions that act like protective shells, and they can be long-lived (sometimes up to several months) [15]. In contrast, adult males are winged and mobile, but they are tiny and fragile and have a limited life span of a few days at most [15,16]. Such extreme sexual dimorphism exposes the immobile females to high levels of competition over mate resources represented by the fragile and short-lived males [17], which may lead to the evolution of reproductive systems that depend less on males [14]. In fact, males are either very rare or unknown in many scale insects, particularly in mealybugs: 10% of the species reproduce either partially or completely by parthenogenesis [18], a form of asexual reproduction in which embryos develop without fertilization.

The pineapple mealybug, *Dysmicoccus brevipes* (Cockerell), is one such parthenogenetic species. It is a known vector of pineapple wilt–associated viruses, which severely reduce pineapple yields [19], and this species also attacks many other agricultural crops [20,21]. *Dysmicoccus brevipes* was first described in Jamaica and is apparently native to the New World [22], but it now has a cosmopolitan distribution associated with pineapple transportation and cultivation. Many previous studies [23–27] conducted in a variety of areas indicated that this species reproduces by obligate apomictic thelytokous parthenogenesis [18], although the presence of adult males has been reported [22]. At least on the Hawaiian Islands, only parthenogenetic females are found; males have yet to be discovered and are probably not present [22]. These findings suggest that both sexual and asexual reproductive systems are included in the same or a very closely related lineage, and thus *D*. *brevipes* may provide a good opportunity to examine the evolutionary consequences of competition between sexual and asexual lineages. However, little is known about the distributions of sexual and asexual *D*. *brevipes* lineages as well as their phylogenetic relationship.

Therefore, in the present study we first surveyed the occurrences and frequencies of sexual and asexual individuals in *D*. *brevipes* populations sampled on two islands in southwestern Japan. The sexual and asexual lineages co-exist on one of the islands, whereas only the asexual lineage is present in the other. We then measured their fecundity and growth rates under laboratory conditions to examine the basic elements of competition between the lineages. Finally, we attempted to elucidate their molecular phylogeny by using partial sequences of genomes of mitochondria, nuclei, and the primary endosymbiotic bacterium, *Candidatus* Tremblaya princeps, which is ubiquitously present in the cytoplasm of mealybug bacteriocytes and is maternally inherited [28,29]. Based on these results, we discussed the potential factors that may have influenced the maintenance and/or competitive elimination of sexual/asexual reproductive systems in this unique insect.

## Materials and Methods

### Insects

Gravid ovoviviparous females of *D*. *brevipes* were collected in pineapple fields of Okinawa Prefectural Agricultural Research Center (OPARC) on Okinawa Island (26.6°N, 128.0°E; Nago city, Okinawa Prefecture, Japan) and Ishigaki Island (24.4°N, 124.2°E; Ishigaki city, Okinawa Prefecture, Japan) with permissions and helps of Drs. S. Ohno, I. Yonaha, K. Yonamine, and C. Moromizato (OPARC). Each mealybug was collected from a different colony in order to avoid sampling bias. They were placed individually in a tight-sealed laboratory dish (5.5 cm diameter × 2.5 cm height) in a rearing room (16:8 light:dark; 23°C; 50% relative humidity), fed with a germinated broad bean plant, allowed to produce offspring for 1 week, and then soaked in ethanol and stored at –20°C for DNA extraction and sequencing, as described below. The offspring were transferred to a larger tight-sealed laboratory dish (9.0 cm diameter × 3.0 cm height) with germinated broad bean plants and reared as a brood. For sexual mealybugs, the adults that emerged were allowed to copulate for 1 day. Pregnant females were transferred to a new dish with fresh food.

### Assessment of developmental and reproductive performances

Development times, pre-parturition durations, parturition durations, and numbers of offspring were assessed using laboratory-reared mealybugs under the conditions described above. For the assessment of development times, freshly born nymphs were transferred individually to a germinated broad bean plant placed in a tight-sealed dish and were monitored daily. Males made cocoons and became pupae after three molts, whereas females matured to adulthood after three molts without pupal metamorphosis. To assess reproductive performance parameters, a fresh adult female just after the final molt was placed individually in a tight-sealed dish and fed with a germinated broad bean plant. Each female of the sexual lineages was housed with one adult male that was randomly chosen from stock culture, and copulation was confirmed visually. Offspring borne by each female were counted every day until the female stopped parturition.

### DNA extraction and sequencing

Total genomic DNA was extracted from the whole body of mealybug females using a DNeasy Blood and Tissue Kit (Qiagen, Tokyo, Japan). The mitochondrial cytochrome oxidase subunit I (*CO1*) gene (ca. 1.4 kbp) and the endosymbiont bacterial RNA polymerase β subunit (*rpoB*; ca. 1.2 kbp) gene were subsequently amplified by PCR with *Taq* DNA polymerase (Takara Ex Taq; Takara Bio Inc., Shiga, Japan; 0.5 units in 20 μl of reaction mixture) and a set of primers (0.5 μM in final concentration), namely *CO1*: 5′-tatttaatatttggattttgatcagg-3′ (forward) and 5′-caatgcatattattctgccatatt-3′ (reverse); *rpoB*: 5′-cgacgtggatgacatgagta-3′ (forward) and 5′-gatattctgcccacgatgat-3′ (reverse). We used a temperature profile of 35 cycles of 95°C for 1 min, 55°C for 1 min, and 72°C for 1 min. The PCR products were purified using a PCR Purification Kit (Qiagen) and served as a template for a direct sequencing reaction using a BigDye Terminator Kit ver. 3.1 (Applied Biosystems, Tokyo, Japan). The following internal sequencing primers were used in the sequencing reaction: *CO1*: 5′-ttttaccaggttttggagctat-3′ (forward) and 5′-aaaaattttaattcttgttggaatagc-3′ (reverse); *rpoB*: 5′-gtacacccctcggaggtaat-3′ (forward) and 5′-gcttacgtggatagcagcat-3′ (reverse). The sequencing was performed using a genetic analyzer (ABI 3130; Applied Biosystems). In addition, the internal transcribed spacer between ribosomal RNA genes (ITS2, ca. 0.7 kbp) of the nuclear genome was partially amplified by the primers ITS-M-F (5′-ctcgtgaccaaagagtcctg-3′) and ITS-M-R (5′-tgcttaagttcagcgggtag-3′) [30] and sequenced. For comparisons, *CO1* and *rpoB* were amplified and sequenced from the genomic DNA of other mealybug species: *Planococcus citri*, *Planococcus minor*, *Planococcus kraunhiae*, *Pseudococcus comstocki*, *Pseudococcus cryptus*, *Crisicoccus matsumotoi*, and *Dysmicoccus neobrevipes*. The DNA solutions and the voucher specimens are stored in the National Institute for Agro-Environmental Sciences (Tsukuba, Japan).

### Phylogenetic analyses

The gene sequences were aligned using ClustalX software [31]. The final alignment was inspected and corrected manually, and only unambiguous nucleotide sites were used for analyses. Maximum likelihood trees with bootstrap values based on 1000 resamplings were constructed using TreeFinder [32]. ModelTest 3.7 [33] and PAUP* were used to determine appropriate evolutionary models by Akaike information criterion with a correction for finite sample sizes (AICc); the TIM + G model, TVM + I model, and TVM + G model were selected for the tree searches of *CO1*, *rpoB*, and ITS2, respectively.

## Results

### Reproductive systems of the pineapple mealybug

**Fig 2:**
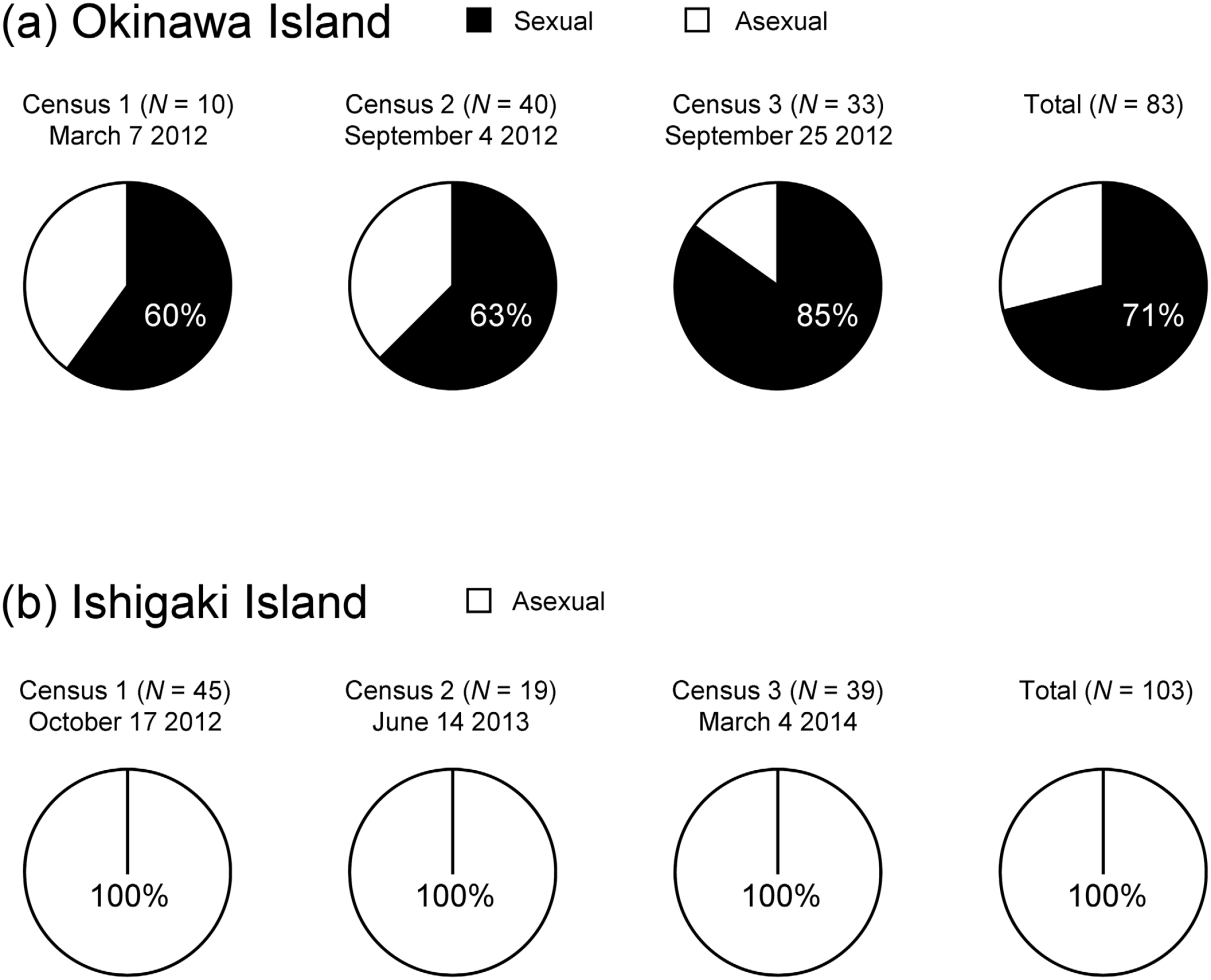
Frequencies of sexual and asexual individuals of *Dysmicoccus brevipes* from Okinawa (a) and Ishigaki (b).

Two completely different reproductive systems were observed in the pineapple mealybugs collected on Okinawa: 59 wild females produced both male and female offspring (29.3% female offspring on average), whereas 24 females produced only female offspring with no males (Fig 2). Females from the female-only broods produced only female offspring without copulation, indicating thelytokous parthenogenesis. Females from the broods with both sexes were able to produce both male and female offspring only after copulation and never produced offspring without copulation, indicating sexual reproduction. No copulations were observed in pairs of a male and a female of an asexual brood (>10 pairs examined), whereas copulation was observed within a few minutes when a male was placed with a virgin female of a sexual brood. As far as we observed, both of the reproductive systems were maternally hereditable and completely obligatory. The sex ratios of offspring in some matrilines are shown in S1 Fig.

### Co-existence of sexually and asexually reproducing lineages

Both sexually and asexually reproducing individuals were consistently observed in three censuses in a pineapple field on Okinawa; the proportion accounted for by the sexual lineage was always predominant (60–85%) and, in total, significantly deviated from the expectation of 1:1 co-existence of the two lineages (*χ*^*2*^ = 14.8, df = 1, *P* < 0.001; Fig 2). On the other hand, only an asexual lineage was found in a pineapple field on Ishigaki; the 103 matrilines collected during three censuses were examined but no sexual reproduction was observed.

Performances with respect to the development of the two lineages of mealybugs from Okinawa were generally similar except for the sex ratios in their offspring. However, the pre-parturition duration (i.e., the time until the adult females started producing nymphs) was significantly shorter in the sexual lineage (Mann-Whitney *U*-test, *P* < 0.001; Table 1). The detailed data for developmental and reproductive performances are shown in S1 Table.

**Table 1:** Developmental and reproductive performances (mean ± SE) of the sexually and asexually reproducing lineages of *Dysmicoccus brevipes* on Okinawa Island.

Lineage	Sex	Development time (day)	Pre- parturition duration (day)	Parturition duration (day)	Number of offspring (% females)
Sexual	Female (N = 33)	25.4 ± 0.35	21.9 ± 0.26	28.3 ± 1.81	114.8 ± 6.90 (46.1 ± 2.04%)
	Male (N = 15)	24.3 ± 0.32	-	-	-
Asexual	Female (N = 21)	24.1 ± 0.64	34.8 ± 1.22	22.3 ± 0.69	107.7 ± 9.11 (100%)

### Molecular characterization and phylogeny of sexual and asexual lineages

Partial sequences of mitochondrial and nuclear genomes, as well as the primary endosymbiotic bacterial genome, were examined individually in 40 mealybugs collected from Okinawa (32 sexual females and 8 asexual females) and 45 mealybugs collected from Ishigaki (all asexual).

**Fig 3:**
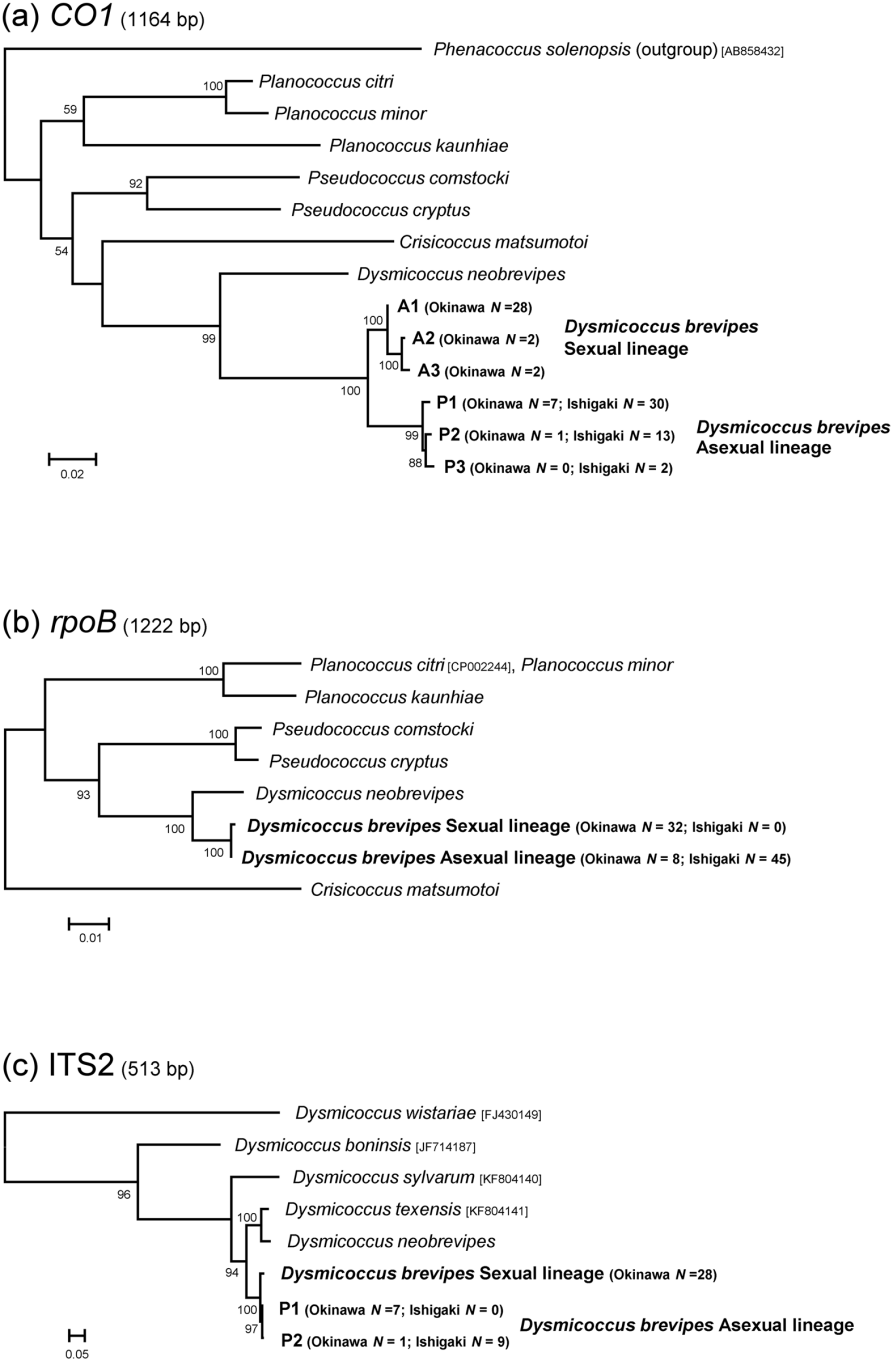
Phylogeny of the sexual and asexual lineages based on partial sequences of mitochondria (*CO1*; a), an intracellular symbiont (*rpoB*; b), and the nuclear (ITS2; c) genome. The trees were constructed by the maximum likelihood method using unambiguously aligned nucleotide sites. The trees of *rpoB* and ITS2 are rooted on each midpoint. The bootstrap values (>50%) obtained from 1000 resamplings are given at the nodes. Sequences of the taxa with the DDBJ/EMBL/GenBank accession numbers in brackets were obtained from the database.

Six haplotypes were found based on the mitochondrial *CO1* sequences (1449 bp). These haplotypes were clearly divided into two groups corresponding to the sexual lineage and the asexual lineage (Fig 3a). Among the haplotypes of the sexual lineage, haplotype A1 was predominant (87.5%). In the asexual lineage, haplotype P1 was similarly predominant both on Okinawa (87.5%) and Ishigaki (66.7%). Each haplotype represented a group that was the most closely related among the *CO1* sequences of all mealybug species examined and clustered into clades with high bootstrap values (Fig 3a). A total of 35 nucleotide sites were substituted (2.4%; 16 transitions and 19 transversions involving eight amino acid substitutions) between haplotypes A1 and P1.

Partial sequences of a single-copy protein-coding gene (*rpoB*; 1350 bp) of the primary endosymbiont, *Candidatus* Tremblaya princeps, harbored in the cytoplasm of mealybugs were very similar in the two lineages, but one nucleotide mutation involving an amino acid substitution was found. No differences were discovered in the sequences of the samples examined within each lineage. Like the *CO1* sequences, the *rpoB* sequence-based phylogenetic tree showed that the two lineages of *D*. *brevipes* were closely allied and formed a reliable clade (Fig 3b).

The ITS2 region in the mealybug nuclear genome was successfully determined for 36 and 9 individuals collected from Okinawa and Ishigaki, respectively. Sequences of the PCR products from the other samples could not be determined by direct sequencing, probably because of intra-individual variations of the ITS2 region. The ITS2 sequences also included a fixed difference: one sequence was obtained from the samples of the sexual lineage (688 bp) and two sequences from the asexual lineage (692 bp). The two sequences of the asexual lineage had only one transition mutation, whereas two gaps and 14 nucleotide substitutions (7 transitions and 7 transversions) were found between the sexual lineage and the asexual lineage. Phylogenetic analysis of the ITS2 sequences of the genus *Dysmicoccus* using the maximum likelihood method demonstrated that the two lineages of *D*. *brevipes* were the most closely related of any of the mealybugs examined and formed a monophyletic clade (Fig 3c).

In the *CO1* tree, we used the sequence from *Phenacoccus solenopsis*, which belongs to a different subfamily (Phenacoccinae), as the outgroup to show the phylogenetic position of *D*. *brevipes* (Pseudococcinae) among mealybug species. No reliable alignment results were generated in analyses of *rpoB* and ITS2 when sequences of Phenacoccinae samples were used, and therefore the trees rooted on each midpoint were shown to demonstrate only the phylogenetic relationship of the two lineages of *D*. *brevipes*. The partial DNA sequences determined in the present study were deposited in the DDBJ database with the accession numbers LC121493-LC121517.

## Discussion

The present study demonstrated that distinct reproductive modes—sexual and asexual—coexist in a local, non-native population of the pineapple mealybug, *D*. *brevipes* (Figs 1 and 2). The mealybugs with the distinct reproductive modes are behaviorally and genetically isolated and are likely to represent diverged lineages, although there are no morphological diagnostic features. The molecular genotyping data support this idea. DNA sequences of mitochondrial and nuclear genomes as well as the endosymbiotic bacterial genome were closely related between the lineages, but clearly separated based on the reproductive mode of the mealybug (Fig 3). This study offers a unique example to investigate the evolution and ecology of sexual and asexual reproduction.

Several haplotypes with less than 0.5% nucleotide substitutions of the mitochondrial *CO1* gene were discovered in each lineage, although the haplotypes A1 and P1 were predominant in the sexual and asexual lineages, respectively (Fig 3a). Based on the previously reported pairwise divergence rate of *CO1* sequences in several arthropod groups, which was found to cluster approximately 1.5% per million years [34,35], the *CO1* genes of the two lineages are estimated to have diverged about 1.3 million years ago. The difference between the mitochondrial DNA sequences of the two lineages was relatively small compared to intraspecific variations in some insect taxa [36], suggesting that the lineages are very closely related, although they are reproductively isolated and genetically different. This finding is consistent with the partial sequences of ITS2 in the nuclear genome, with its higher evolutionary rate, and the endosymbiotic bacterial genome (*rpoB*); the two lineages were the most closely related and formed a monophyletic group in the genus *Dysmicoccus* (Fig 3).

Other members of the genus *Dysmicoccus* employ sexual reproduction [37,38] and obligate thelytokous parthenogenesis is reported only in *D*. *brevipes* in this genus [18], indicating that the common ancestor of the two lineages of *D*. *brevipes* would have reproduced sexually. Scale insects display a remarkable variety of genetic systems for sex determination and reproduction including parthenogenesis [13], and the ancestral system in mealybugs is assumed to have been sexual reproduction with males, the paternally derived genome of which is deactivated through heterochromatinization (paternal genome elimination: PGE) [39]. Two types of parthenogenetic reproduction, obligate apomictic thelytoky and obligate automictic thelytoky, are found in mealybugs and these systems are likely to have evolved independently multiple times from the PGE system [18]. In the PGE system with sexual reproduction, paternal genomes are not included during spermatogenesis and thus are not inherited, and therefore a female-biased sex ratio in offspring is favored by a male parent [13,14]. Moreover, female scale insects are more robust than males with respect to physiology and morphology [15]. In addition, females benefit more than males from mutualistic relationships with ants, which care for and guard females in exchange for payment of honeydew [13,15]. These features could potentially drive mealybugs to evolve parthenogenetic reproduction that does not depend on males.

It is therefore difficult to understand how sexual reproduction with the PGE genomic system has been stable and prevalent in scale insects, including some lineages of *D*. *brevipes* despite strong selective pressures against fragile males. The present case on Okinawa clearly shows that the sexual lineage can simultaneously and sympatrically exist with and even dominate the asexual lineage (Fig 2) in the face of habitat and resource competition, which is considered to be severe for the nearly immobile scale insects. This suggests that advantages offered by sexual reproduction compensate for the cost of males on Okinawa. One of the advantages may be associated with a significantly shorter pre-parturition period in the sexual lineage, although it does not directly lead to better intrinsic population growth rate, because only a portion of the offspring (≈46%) are female and thus produce offspring (Table 1). Copulation can be a trigger for ovarian maturation and oviposition in insects [40–42], which may explain the difference between pre-parturition duration in the two reproductive systems. Advanced parturition would reduce the risk of female mealybugs encountering natural enemies or biotic/abiotic accidents, leading to an increased probability of reproductive success under natural conditions. Because our assessment of developmental and reproductive performances made use of a substituted diet (germinated broad bean plants) under laboratory conditions, we may have underestimated the differences between the lineages in nature. Moreover, the two lineages may have diverged enough to have some different ecological niches, although they occur simultaneously and sympatrically in pineapple fields. Further comparisons and investigations of various host plants including pineapples and other crops are necessary to elucidate their degree of competition.

The two reproductive lineages co-exist in a part of a non-native population (Okinawa), whereas only the asexual lineage is found on another island (Ishigaki) in the same archipelago (Fig 2). One simple explanation for this difference is that different colonies of the mealybug lineages may have been introduced in association with pineapples; that is, both lineages reached Okinawa but only the asexual lineage reached Ishigaki. This is unlikely, however, because these two islands belong to and are managed by the same prefectural government (Okinawa Prefecture); therefore, pineapple transportation and cultivation are assumed to have similar histories on the two islands. Pineapple cultivation started in the 1920s and 1930s in Japan [43]. The mealybugs are reported to have first invaded a district including Ishigaki along with imported pineapple plants from Taiwan at an early stage of cultivation, and they subsequently dispersed to Okinawa via pineapple seedling exchange [44]. If this scenario is true, it implies that the asexual lineage has outcompeted the sexual lineage on Ishigaki, their original area of invasion, where no sexual individuals are currently observed.

Thus, an alternative hypothesis is that the mealybugs on Okinawa and Ishigaki may have been exposed to different environmental stresses, such as those imposed by a different climate or natural enemy fauna, which would lead to differences in relative fitness of the sexual and asexual lineages and in frequencies of the sexual and asexual lineages on each island. For example, climate conditions in winter are more severe on Okinawa (winter daily minimum temperatures ≈13°C) than on Ishigaki (≈17°C) [45]. Lower temperatures would impose acquisition of cold hardiness on this tropical insect and the genetic diversity offered by sexual reproduction might have been favored on Okinawa, whereas Ishigaki has a relatively moderate winter climate and asexual mealybugs with higher growth rates might have outcompeted their sexual relatives. Further surveys of the two reproductive lineages of this unusual insect across a broad range of native and non-native populations and detailed investigations of their habitats may provide more insight into the evolution and ecology of the sexual and asexual reproductive systems in scale insects.

## Supporting Information

S1 Fig Sex ratios (female:male) of offspring in some matrilines of the sexual and asexual lineages of *Dysmicoccus brevipes*. Each box indicates offspring borne by a single female.
(PDF)

S1 Table Developmental and reproductive performances in the sexual and asexual lineages of *Dysmicoccus brevipes*. (PDF)

## References

[1] Hamilton WD, Axelrod R, Tanese R. Sexual reproduction as an adaptation to resist parasites (a review). Proc Natl Acad Sci USA. 1990;87: 3566–3573. 10.1073/pnas.87.9.3566 2185476 PMC53943

[2] Kondrashov AS. Deleterious mutations and the evolution of sexual reproduction. Nature. 1988;334: 435–440. 10.1038/336435a0 3057385

[3] Lively CM. A review of Red Queen models for the persistence of obligate sexual reproduction. J Hered. 2010;101(Suppl 1): S13–S20. 10.1093/jhered/esq010 20421322

[4] Maynard Smith J. The evolution of sex. New York: Cambridge University Press; 1978.

[5] Meirmans S. The evolution of the problem of sex In: Schoen I, Martens K, van Dijk P, editors. The evolutionary biology of parthenogenesis. Dordrecht: Springer; 2009 pp. 21–46. 10.1007/978-90-481-2770-2_2

[6] Meirmans S, Meirmans PG, Kirkendall LR. The costs of sex: Facing real-world complexities. Q Rev Biol. 2012;87: 19–40. 10.1086/663945 22518931

[7] Otto SP. The evolutionary enigma of sex. Am Nat. 2009;174(Suppl 1): S1–S14. 10.1086/599084 19441962

[8] Otto SP, Lenormand T. Resolving the paradox of sex and recombination. Nat Rev Genet. 2002;3: 252–261. 10.1038/nrg761 11967550

[9] West SA, Lively CM, Read AF. A pluralist approach to sex and recombination. J Evol Biol. 1999;12: 1003–1012. 10.1046/j.1420-9101.1999.00119.x

[10] Williams GC. Sex and evolution. Princeton: Princeton University Press; 1975.

[11] Schwander T, Crespi BJ. Molecular evidence for ancient asexuality in *Timema* stick insects. Curr Biol. 2011;21: 1–6. 21683598 10.1016/j.cub.2011.05.026

[12] van der Kooi C, Schwander T. Evolution of asexuality via different mechanisms in grass thrips (thysanoptera: Aptinothrips). Evolution 2014;68: 1883–1893. 10.1111/evo.12402 24627993

[13] Ross L, Pen I, Shuker DM. Genomic conflict in scale insects: the causes and consequences of bizarre genetic systems. Biol Rev. 2010;85: 807–828. 10.1111/j.1469-185X.2010.00127.x 20233171

[14] Ross L, Shuker DM, Normark BB, Pen I. The role of endosymbionts in the evolution of haploid-male genetic systems in scale insects (Coccoidea). Ecol Evol. 2012;2: 1071–1081. 10.1002/ece3.222 22837851 PMC3399172

[15] Ross L, Shuker DM. Scale insects. Curr Biol. 2009;19: R184–R186. 10.1016/j.cub.2008.12.023 19278625

[16] Dunkelbulm E. Scale insects In: Hardie J, Minks AK, editors. Pheromones of non-lepidopteran insects associated with agricultural plants. Oxfordshire: CAB International; 1999 pp. 251–276.

[17] Tabata J, Narai Y, Sawamura N, Hiradate S, Sugie H. A new class of mealybug pheromones: a hemiterpene ester in the sex pheromone of *Crisicoccus matsumotoi*. Naturwissenschaften. 2012;99: 567–574. 10.1007/s00114-012-0935-z 22751867

[18] Nur U. Parthenogenesis in Coccids (Homoptera). Am Zool. 1971;11: 301–308.

[19] Sether DM, Ullman DE, Hu JS. Transmission of pineapple mealybug wilt-associated virus by two species of mealybug (*Dysmicoccus* spp.). Phytopathology. 1998;88: 1224–1230. 10.1094/PHYTO.1998.88.11.1224 18944858

[20] Ben-Dov Y. A systematic catalogue of the mealybugs of the world (Insecta: Homoptera: Coccoidae: Pseudococcidae and Putoidae) with data on geographical distribution, host plants, biology and economic importance. Andover: Intercept; 1994

[21] García M, Denno B, Miller DR, Miller GL, Ben-Dov Y, Hardy NB. ScaleNet: A literature-based model of scale insect biology and systematics. 2016;1: 27 Available: http://scalenet.info/. 10.1093/database/bav118 PMC4747323 26861659

[22] Beardsley JW. Notes on the pineapple mealybug complex, with descriptions of two new species (Homoptera: Pseudococcidae). Proc Hawaiian Entomol Soc. 1965;19: 55–68.

[23] Ito K. Studies on the life history of the pineapple mealybug, *Pseudococcus* brevipes (Ckll.). J Econ Entomol 1938;31: 291–298. 10.1093/jee/31.2.291

[24] Carter W. The geographical distribution of mealybug wilt with notes on some other insect pests of pineapple. J Econ Entomol 1942;35: 10–15. 10.1093/jee/35.1.10

[25] Kawai S. Scale insects of Japan in colors. Tokyo: Zenkoku-Noson-Kyouiku-Kyoukai; 1980.

[26] Rohrbach KG, Beardsley JW, German TL, Reimer NJ, Sanford WG. Mealybug wilt, mealybugs, and ants of pineapple. Plant Dis. 1988;72: 558–565. 10.1094/PD-72-0558

[27] Bertin A, Bortoli LC, Botton M, Parra JRP. Host plant effects on the development, survival, and reproduction of *Dysmicoccus brevipes* (Hemiptera: Pseudococcidae) on grapevines. Ann Entomol Soc Am. 2013;106: 604–609. 10.1603/AN13030

[28] Thao MY, Gullan PJ, Baumann P. Endosymbionts infect the primary (β-Proteobacteria) endosymbionts of mealybugs multiple times and coevolve with their hosts. Appl Environ Microbiol. 2002;68: 3190–3197. 12088994 10.1128/AEM.68.7.3190-3197.2002 PMC126777

[29] von Dohlen CD, Kohler S, Alsop ST, McManus WR. Mealybug beta-proteobacterial endosymbionts contain gamma-proteobacterial symbionts. Nature. 2001;412: 433–436. 10.1038/35086563 11473316

[30] Malausa T, Fenis A, Warot S, Germain J-F, Ris N, Prado E, et al DNA markers to disentangle complexes of cryptic taxa in mealybugs (Hemiptera: Pseudococcidae). J Appl Entomol. 2011;135: 142–155. 10.1111/j.1439-0418.2009.01495.x

[31] Thompson JD, Higgins DG, Gibson TJ. CLUSTAL W: improving the sensitivity of progressive multiple sequence alignment through sequence weighting, position-specific gap penalties and weight matrix choice. Nucleic Acids Res. 1994;11: 4673–4680. 10.1093/nar/22.22.4673 PMC308517 7984417

[32] Jobb G, von Haeseler A, Strimmer K. TREEFINDER: a powerful graphical analysis environment for molecular phylogenetics. BMC Evol Biol. 2004;4: 18 10.1186/1471-2148-4-18 15222900 PMC459214

[33] Posada D, Crandall KA. MODELTEST: testing the model of DNA substitution. Bioinformatics. 1998;14: 817–818. 10.1093/bioinformatics/14.9.817 9918953

[34] Quek SP, Davies SJ, Itino T, Pierce NE. Codiversification in an ant-plant mutualism: stem texture and the evolution of host use in Crematogaster (Formicidae: Myrmicinae) inhabitants of Macaranga (Euphorbiaceae). Evolution. 2004;58: 554–570. 10.1554/03-361 15119439

[35] Ueda S, Quek SP, Itioka T, Inamori K, Sato Y, Murase K, et al An ancient tripartite symbiosis of plants, ants and scale insects. Proc R Soc B. 2008;275: 2319–2326. 10.1098/rspb.2008.0573 18611850 PMC2603224

[36] Vogler AP, Knisley CB, Glueck SB, Hill JM, Desalle R. Using molecular and ecological data to diagnose endangered populations of the puritan tiger beetle *Cicindela puritana*. Mol Ecol. 1993;2: 375–383. 10.1111/j.1365-294X.1993.tb00030.x 7909261

[37] De Alfonso I, Hernandez E, Velazquez Y, Navarro I, Primo J. Identification of the sex pheromone of the mealybug *Dysmicoccus grassii* Leonardi. J Agric Food Chem. 2012;60: 11959–11964. 10.1021/jf304065d 23167613

[38] Tabata J, Ichiki RT. A new lavandulol-related monoterpene in the sex pheromone of the grey pineapple mealybug, *Dysmicoccus neobrevipes*. J Chem Ecol. 2015;41: 194–201. 10.1007/s10886-015-0545-2 25618324

[39] Nur U. Evolution of unusual chromosome systems in scale insects (Coccoidea: Homoptera) In Blackman RL, Hewitt GM, Ashburner M, editors. Insect cytogenetics. Oxford: Blackwell; 1980 pp. 97–118.

[40] Thailayil J, Magnusson K, Godfray HCJ, Crisanti A, Catteruccia F. Spermless males elicit large-scale female responses to mating in the malaria mosquito *Anopheles gambiae*. Proc Natl Acad Sci USA. 2011;108: 13677–13681. 10.1073/pnas.1104738108 21825136 PMC3158155

[41] Xu J, Wang Q. Seminal fluid reduces female longevity and stimulates egg production and sperm trigger oviposition in a moth. J Insect Physiol. 2011;57: 385–390. 10.1016/j.jinsphys.2010.12.006 21172356

[42] Loher W, Ganjian I, Kubo I, Stanley-Samuelson D, Tobe SS. Prostaglandins: Their role in egg-laying of the cricket *Teleogryllus commodus*. Proc Natl Acad Sci USA. 1981;78: 7835–7838. 10.1073/pnas.78.12.7835 16593135 PMC349366

[43] Shoda M, Urasaki N, Sakiyama S, Terakami S, Hosaka F, Shigeta N, et al DNA profiling of pineapple cultivars in Japan discriminated by SSR markers. Breed Sci. 2012;62: 352–359. 10.1270/jsbbs.62.352 23341750 PMC3528333

[44] Kohama T, Takehara K. Exotic insects in Okinawa. Bull Okinawa Pref Mus. 2002;28: 55–59.

[45] Tables of monthly climate statistics. Japan Meteorological Agency. 2016;4: 11. Available: http://www.data.jma.go.jp/obd/stats/data/en/smp/.

