# Supplementary figures and images for: Sexual versus Asexual Reproduction: Distinct Outcomes in Relative Abundance of Parthenogenetic Mealybugs following Recent Colonization
Source: PLoS One. 2016 Jun 20;11(6):e0156587. doi: 10.1371/journal.pone.0156587 (PMC4920589; doi:10.1371/journal.pone.0156587)

(a) Sexual lineage

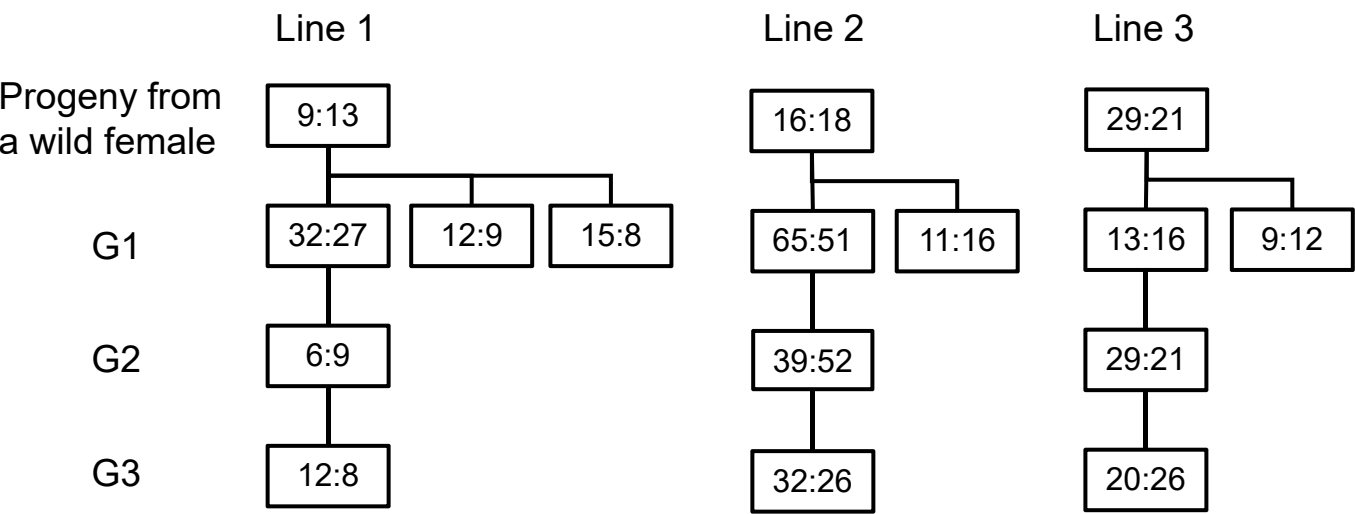

(b) Asexual lineage

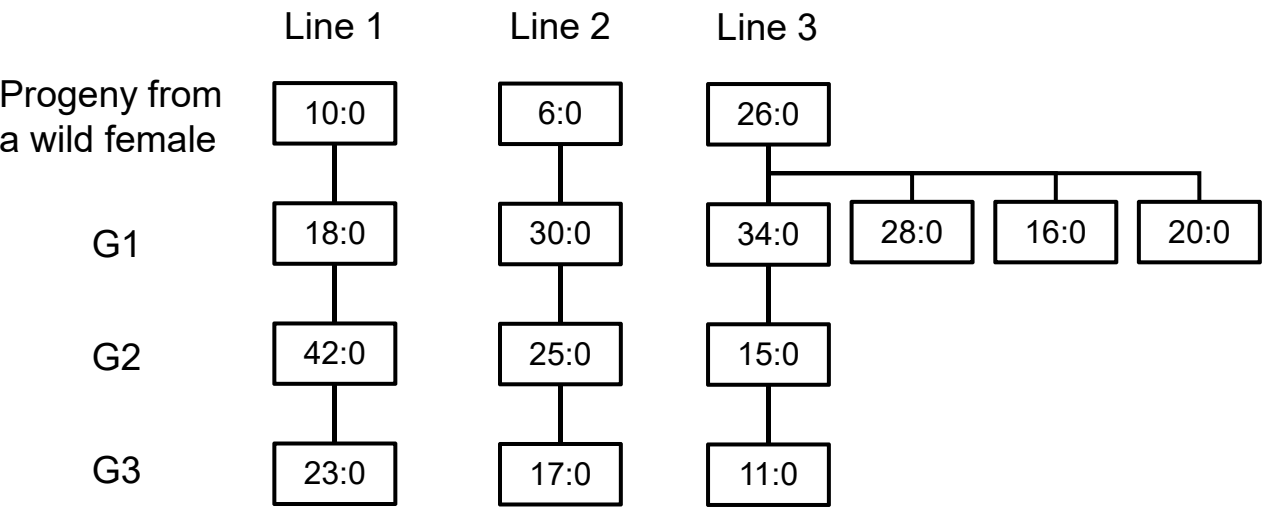

Supplement: S1 Fig — Each box indicates offspring borne by a single female. (PDF) [file pone.0156587.s001.pdf]
