# Supplementary material for: Sexual versus Asexual Reproduction: Distinct Outcomes in Relative Abundance of Parthenogenetic Mealybugs following Recent Colonization
Source: PLoS One. 2016 Jun 20;11(6):e0156587. doi: 10.1371/journal.pone.0156587 (PMC4920589; doi:10.1371/journal.pone.0156587)
Supplement: S1 Table — (PDF) [file pone.0156587.s002.pdf]

S1 Table. Performances in development and reproduction of the sexual and the asexual lineages of *Dysmicoccus brevipes*.

Code	Lineage	Sex	Development time (day)	Pre- parturition duration (day)	Parturition duration (day)	Number of offspring (% females)
SF-01	Sexual	Female	28	22	18	71 (40.8)
SF-02	Sexual	Female	28	21	30	111 (54.0)
SF-03	Sexual	Female	27	23	48	197 (33.5)
SF-04	Sexual	Female	27	23	18	119 (65.5)
SF-05	Sexual	Female	21	27	14	61 (42.6)
SF-06	Sexual	Female	27	22	22	69 (59.4)
SF-07	Sexual	Female	27	22	48	221 (47.1)
SF-08	Sexual	Female	27	22	30	145 (52.4)
SF-09	Sexual	Female	27	22	22	48 (60.4)
SF-10	Sexual	Female	27	22	18	77 (33.8)
SF-11	Sexual	Female	27	22	30	112 (41.1)
SF-12	Sexual	Female	27	22	47	104 (21.2)
SF-13	Sexual	Female	27	22	48	108 (45.4)
SF-14	Sexual	Female	28	22	44	120 (49.2)
SF-15	Sexual	Female	28	22	44	98 (22.4)
SF-16	Sexual	Female	23	16	22	75 (42.7)
SF-17	Sexual	Female	23	22	47	69 (43.5)
SF-18	Sexual	Female	24	22	29	165 (53.9)
SF-19	Sexual	Female	23	19	22	109 (38.5)
SF-20	Sexual	Female	23	22	26	155 (45.8)
SF-21	Sexual	Female	23	22	26	142 (40.8)
SF-22	Sexual	Female	23	22	27	121 (44.6)
SF-23	Sexual	Female	23	22	23	103 (42.7)
SF-24	Sexual	Female	23	22	20	107 (43.9)
SF-25	Sexual	Female	23	22	17	98 (59.2)
SF-26	Sexual	Female	26	22	23	199 (37.2)
SF-27	Sexual	Female	26	22	26	131 (46.6)
SF-28	Sexual	Female	26	22	29	105 (41.9)
SF-29	Sexual	Female	26	22	22	89 (64.0)

SF-30	Sexual	Female	26	22	21	134 (40.3)
SF-31	Sexual	Female	25	22	24	103 (54.4)
SF-32	Sexual	Female	25	22	27	124 (77.4)
SF-33	Sexual	Female	25	22	22	99 (36.4)
SM-01	Sexual	Male	23	-	-	-
SM-02	Sexual	Male	24	-	-	-
SM-03	Sexual	Male	24	-	-	-
SM-04	Sexual	Male	24	-	-	-
SM-05	Sexual	Male	25	-	-	-
SM-06	Sexual	Male	27	-	-	-
SM-07	Sexual	Male	27	-	-	-
SM-08	Sexual	Male	24	-	-	-
SM-09	Sexual	Male	23	-	-	-
SM-10	Sexual	Male	23	-	-	-
SM-11	Sexual	Male	24	-	-	-
SM-12	Sexual	Male	24	-	-	-
SM-13	Sexual	Male	25	-	-	-
SM-14	Sexual	Male	24	-	-	-
SM-15	Sexual	Male	24	-	-	-
PF-01	Asexual	Female	21	33	20	148 (100)
PF-02	Asexual	Female	25	44	24	124 (100)
PF-03	Asexual	Female	25	35	24	97 (100)
PF-04	Asexual	Female	22	35	24	69 (100)
PF-05	Asexual	Female	23	42	22	21 (100)
PF-06	Asexual	Female	22	30	16	145 (100)
PF-07	Asexual	Female	25	37	25	89 (100)
PF-08	Asexual	Female	21	42	26	154 (100)
PF-09	Asexual	Female	21	44	19	149 (100)
PF-10	Asexual	Female	25	33	20	51 (100)
PF-11	Asexual	Female	23	30	24	154 (100)
PF-12	Asexual	Female	21	33	24	79 (100)
PF-13	Asexual	Female	29	34	24	139 (100)
PF-14	Asexual	Female	25	27	22	119 (100)
PF-15	Asexual	Female	26	34	16	62 (100)
PF-16	Asexual	Female	19	42	25	78 (100)

PF-17	Asexual	Female	30	32	26	149 (100)
PF-18	Asexual	Female	28	22	19	55 (100)
PF-19	Asexual	Female	28	33	19	148 (100)
PF-20	Asexual	Female	25	35	22	124 (100)
PF-21	Asexual	Female	23	34	27	149 (100)
